# Norovirus Disease in the United States

**DOI:** 10.3201/eid1908.130465

**Published:** 2013-08

**Authors:** Aron J. Hall, Ben A. Lopman, Daniel C. Payne, Manish M. Patel, Paul A. Gastañaduy, Jan Vinjé, Umesh D. Parashar

**Affiliations:** Centers for Disease Control and Prevention, Atlanta, Georgia, USA

**Keywords:** norovirus, viruses, incidence, norovirus disease, epidemic acute gastroenteritis, United States

## Abstract

Although recognized as the leading cause of epidemic acute gastroenteritis across all age groups, norovirus has remained poorly characterized with respect to its endemic disease incidence. Use of different methods, including attributable proportion extrapolation, population-based surveillance, and indirect modeling, in several recent studies has considerably improved norovirus disease incidence estimates for the United States. Norovirus causes an average of 570–800 deaths, 56,000–71,000 hospitalizations, 400,000 emergency department visits, 1.7–1.9 million outpatient visits, and 19–21 million total illnesses per year. Persons >65 years of age are at greatest risk for norovirus-associated death, and children <5 years of age have the highest rates of norovirus-associated medical care visits. Endemic norovirus disease occurs year round but exhibits a pronounced winter peak and increases by ≤50% during years in which pandemic strains emerge. These findings support continued development and targeting of appropriate interventions, including vaccines, for norovirus disease.

Recognition of the public health impact of noroviruses has increased in recent years, driven largely by an abundance of reported outbreaks. A systematic literature review identified >900 published reports of laboratory-confirmed norovirus outbreaks during 1993–2011 (*1*). In contrast, studies assessing endemic norovirus disease are limited primarily to etiologic studies of acute gastroenteritis among children seeking medical care (*2*). Such prevalence studies provide valuable insights into the role of norovirus among patients with acute gastroenteritis. However, robust assessment of the norovirus disease burden, which herein refers to the annual number of illnesses and associated outcomes, requires population-based incidence estimates, ideally from national or nationally representative surveillance. However, there are several challenges to generating such estimates for norovirus in the United States, including lack of a widely used, rapid, and sensitive clinical assay; no public health reporting requirement for individual cases; low health care–seeking rates of patients with acute gastroenteritis; and poor sensitivity of norovirus-specific codes in national administrative databases (*3*).

Before 2008, only 1 published report estimated the burden of norovirus disease in the United States (*4*). In that report, as part of a broader effort to estimate the US burden of foodborne disease, Mead et al. generated pathogen-specific estimates of illnesses, hospitalizations, and deaths, and they estimated the fraction of these outcomes caused by foodborne disease transmission. Annual norovirus-associated illnesses (23 million), hospitalizations (50,000), and deaths (310) were based on extrapolation of the norovirus-attributable proportion from a single community-based study in the Netherlands and applied to the US all-cause acute gastroenteritis incidence from the National Hospital Discharge Survey (NHDS) and the first Population Survey of the Foodborne Diseases Active Surveillance Network (FoodNet). Although limited by the absence of direct US data on norovirus prevalence or incidence, this landmark study demonstrated the predominant role of norovirus in causing foodborne disease and became the most widely cited estimate of the US norovirus disease burden for more than a decade.

We review a collection of subsequently published studies that provided population-based incidence rates of norovirus disease in the United States. By comparing the various methods and triangulating the results, we provide summary estimates of the overall US norovirus disease burden, including specific estimates by age groups and disease outcomes. This review facilitates identification of key groups that would benefit from prevention strategies aimed at controlling norovirus and provides the grist for development of appropriate interventions, including vaccines. Such data are particularly timely and relevant given that a candidate norovirus vaccine is approaching a phase 3 efficacy trial and could potentially be licensed within the next 5–7 years (*5*).

## Methods and Findings

Since publication of the original estimates reported by Mead et al. (*4*), seven studies have been published that provide norovirus disease incidence estimates for the United States (Table 1). These studies can be broadly grouped on the basis of methods into the following categories: attributable proportion extrapolation, laboratory-confirmed population-based surveillance, and indirect attribution from regression modeling.

**Table 1:** Studies estimating incidence of norovirus disease, United States*

Study (reference)	Age group, y	Norovirus-associated outcome	Data source	Data period	Method
Mead et al. (*4*)	All	Deaths, hospitalizations, illnesses	NHDS, FoodNet	1979–1997	Attributable proportion extrapolation
Patel et al. (*2*)	<5	Hospitalizations, ED visits, outpatient visits	NHDS, NAMCS/NHAMCS	1993–2002	Attributable proportion extrapolation
Scallan et al. (*6*)	All	Deaths, hospitalizations, illnesses	NVSS, HCUP-NIS, NHDS, NAMCS/NHAMCS, FoodNet	2000–2006	Attributable proportion extrapolation
Hall et al. (*7*)	All	Outpatient visits, illnesses	HMO passive surveillance, FoodNet	2004–2005	Laboratory-confirmed population-based surveillance
Payne et al. (*8*)	<5	Hospitalizations, ED visits, outpatient visits	NVSN active surveillance, NAMCS/NHAMCS	2008–2010	Laboratory-confirmed population-based surveillance
Hall et al. (*9*)	<5, 5–64, ≥65	Deaths	NVSS	1999–2007	Indirect attribution from regression modeling
Lopman et al. (*10*)	<5, 5–17, 18–64, 65–74, 75–84, ≥85	Hospitalizations	HCUP-NIS	1996–2007	Indirect attribution from regression modeling
Gastañaduy et al. (*11*)	<5, 5–17, 18–64, ≥65	ED visits, outpatient visits	MarketScan	2001–2009	Indirect attribution from regression modeling

*NHDS, National Hospital Discharge Survey; ED, emergency department; NAMCS/NHAMCS, National Ambulatory Medical Care Survey/National Hospital Ambulatory Medical Care Survey; NVSS, National Vital Statistics System; HCUP-NIS, Healthcare Cost Utilization Project Nationwide Inpatient Sample; FoodNet, Foodborne Diseases Active Surveillance Network; HMO, health maintenance organization; NVSN, New Vaccine Surveillance Network.

### Attributable Proportion Extrapolation

Two studies used the available literature to first estimate the proportion of acute gastroenteritis attributable to norovirus then extrapolated that proportion to all-cause acute gastroenteritis incidence. Patel at al. conducted a systematic literature review of the prevalence of norovirus among persons with acute gastroenteritis in the community, outpatient clinics, emergency departments (ED), and hospitals (*2*). Most of the 31 studies included in the review involved hospitalized children and only 1 of the studies was conducted in the United States, underscoring the limited scope of the available literature. Among hospitalizations or ED visits for acute gastroenteritis in children <5 years of age, a pooled proportion of 12% of cases was attributed to norovirus. This norovirus prevalence was then extrapolated to national estimates of acute gastroenteritis in children <5 years of age from NHDS, the National Ambulatory Medical Care Survey (NAMCS), and the National Hospital Ambulatory Care Survey (NHAMCS) (*12*). The resulting annual estimates of 235,000 outpatient visits, 91,000 ED visits, and 23,000 hospitalizations associated with norovirus in US children <5 years of age suggested that norovirus was second only to rotavirus (before implementation of the national rotavirus vaccine program) as a cause of severe acute gastroenteritis in children.

Building upon the approach taken by Mead et al. (*4*), Scallan et al. reported new estimates of the US burden of foodborne disease (*6*). Although specific data sources had improved over the 12 years separating these 2 reports, the methods for estimating norovirus disease remained largely the same, constrained by the dearth of direct testing data in the United States. On the basis of community studies in the United Kingdom and Australia, and the study in the Netherlands used by Mead et al. (*4*), Scallan et al. estimated that 11% of acute gastroenteritis cases were caused by norovirus (*6*). This attributable proportion was then extrapolated to US rates of all-cause acute gastroenteritis from 3 FoodNet population surveys, hospitalizations from 3 databases (NHDS, the Healthcare Cost and Utilization Project Nationwide Inpatient Sample, and NAMCS/NHAMCS), and deaths from the multiple cause-of-death mortality database in the National Vital Statistics System. The resulting norovirus burden estimates across all ages in the United States were slightly lower than those by reported Mead et al. (*4*) in terms of total illnesses (21 million) but higher with respect to hospitalizations (56,000) and deaths (570). The increased estimate for hospitalizations can be explained in part by the fact that Scallan et al. (*6*) extrapolated the norovirus-attributable proportion to all-cause acute gastroenteritis across all age groups, whereas Mead et al. (*4*) applied the norovirus fraction to all-cause acute gastroenteritis only in adults.

### Laboratory-confirmed Population-based Surveillance

In recognition of the need to directly assess the incidence of laboratory-confirmed norovirus infections among acute gastroenteritis patients in the United States, 2 surveillance platforms were leveraged to generate this data. In collaboration with FoodNet and a health maintenance organization (HMO) in the state of Georgia, Hall et al. used a passive sampling strategy in a population-based study of acute gastroenteritis incidence among outpatients (*7*). A random sample of fecal specimens submitted for routine clinical diagnostics (i.e., bacterial culture) were aliquoted for subsequent norovirus testing. Because the samples were derived from a known population catchment based on HMO membership, the resulting norovirus prevalence could then be used to calculate incidence. Health care use rates from 3 FoodNet population surveys were used to scale-up the observed prevalence among patients who submitted fecal specimens to outpatient and community incidence. The resulting adjusted outpatient and community incidence rates for norovirus were 64/10,000 population and 650/10,000 population, respectively (Table 2). If applied to the US population when the samples were collected (2004), these rates correspond to a national estimate of 19 million illnesses and 1.9 million outpatient visits. This total number of norovirus illnesses was within the uncertainty bounds of the estimate of Scallan et al. (*6*) (90% credibility interval 13–31 million) and provided the first estimate based on direct testing of patients with acute gastroenteritis in the United States. Although this passive sampling approach afforded convenience and required relatively little resources, it is potentially subject to substantial bias for 2 reasons. First, only those fecal samples that had a physician order for bacterial culture were tested. Second, the data may have limited generalizability because the study was conducted in a single, relatively young, privately insured population.

**Table 2:** Population-based rates of norovirus disease–associated outcomes across all age groups by outcome*

Outcome	Study (reference)	Country	Rate/10,000 population (uncertainty bounds)†
Deaths	Scallan et al. (*6*)	United States	0.019 (0.011–0.029)
	Hall et al. (*9*)	United States	0.027 (0.023–0.031)
	Verhoef et al. (*13*)	The Netherlands	0.040 (0.020–0.070)
Hospitalizations	Scallan et al. (*6*)	United States	1.9 (1.1–2.9)
	Lopman et al. (*10*)	United States	2.4 (NR)
	Verhoef et al. (*13*)	The Netherlands	1.2 (0.5–2)
Emergency department visits	Gastañaduy et al. (*11*)	United States	13.5 (8.0–18.9)
Outpatient visits	Hall et al. (*7*)	United States	64.0 (36.0–120.0)
	Gastañaduy et al. (*11*)	United States	57.0 (40.0–74)
	Verhoef et al. (*13*)	The Netherlands	92.0 (50.0–150)
	Phillips et al. (*14*)	United Kingdom	54.0 (48.0–60)
	Tam et al. (*15*)	United Kingdom	21.0 (14.0–30)
	Karsten et al. (*16*)	Germany	63.0 (29.0–107)
Total illnesses	Scallan et al. (*6*)	United States	698.0 (430.0–1,028)
	Hall et al. (*7*)	United States	650.0 (370.0–1,200)
	Verhoef et al. (*13*)	The Netherlands	380.0 (264.0–544)
	Phillips et al. (*14*)	United Kingdom	450.0 (380.0–520)
	Tam et al. (*15*)	United Kingdom	470.0 (391.0–565)
	Thomas et al. (*17*)	Canada	1,040.0 (924.0–1,163)

*NR, not reported. †Uncertainty bounds represent 95% CIs for all studies, except for Scallan at al. (*6*), Hall et al. (*7*), and Thomas et al. (*17*), who used 90% credible intervals.

**Figure 1:**
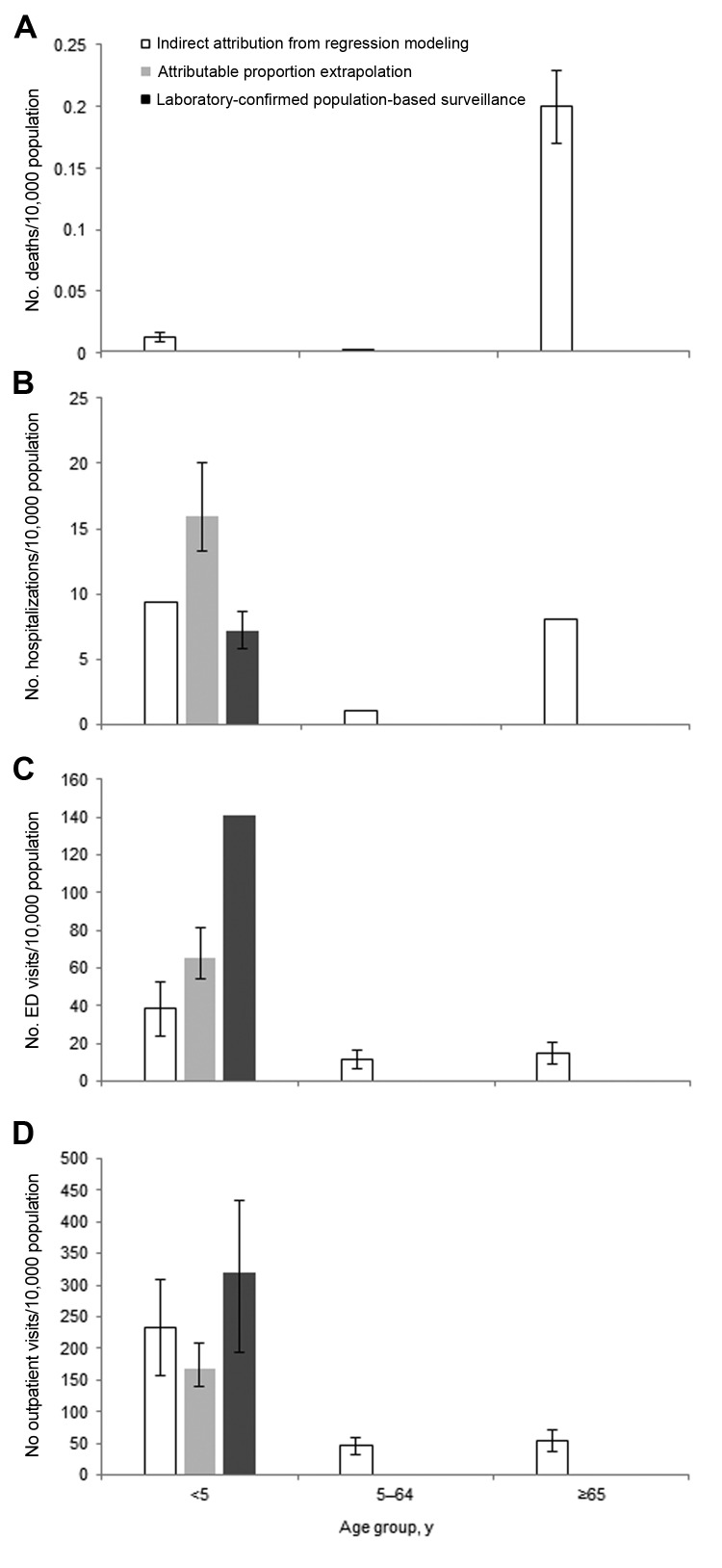
Rates of A) norovirus-associated deaths. B) hospitalizations, C) emergency department (ED) visits, and D) outpatient visits by age group, United States. Data were derived from studies using indirect attribution from regression modeling (*9**–**11*), attributable proportion extrapolation (*2*), and laboratory-confirmed population-based surveillance (*8*). Error bars indicate 95% CIs if reported.

The preferred approach of active surveillance enrollment and laboratory testing of all acute gastroenteritis patients from multiple sites was used in a recent study by Payne et al. from the New Vaccine Surveillance Network (*8*). This network of 3 pediatric hospitals conducted year-round, population-based, active surveillance for hospitalizations, ED visits, and outpatient clinic visits for acute gastroenteritis among children <5 years of age for whom laboratory confirmation of cases was available. Payne et al. reported annual norovirus hospitalization, ED visit, and outpatient visit rates of 7, 141, and 319/10,000 children <5 years of age, respectively, over a 2-year period (*8*). Because the outpatient surveillance in the New Vaccine Surveillance Network used sentinel clinics and was not truly population based, the norovirus outpatient visit rate was based on extrapolation of norovirus prevalence to the all-cause acute gastroenteritis outpatient rates from NAMCS/NHAMCS. Extending these norovirus incidence rates to the ≈20 million US children <5 years of age, Payne et al. estimated 14,000 hospitalizations, 281,000 ED visits, and 627,000 outpatient visits for this age group (*8*). They also reported that the median health care charges for norovirus hospitalizations, ED visits, and outpatient visits were $3,918, $435, and $151, respectively, corresponding to an annual total of $273 million in norovirus-associated treatment costs for US children <5 years of age. Compared with the estimates reported by Patel et al. (*2*) among children <5 years of age, Payne et al. estimated ≈55% fewer hospitalizations but ≈2 times as many ED and outpatient visits (Figure 1). Moreover, Payne et al. reported that norovirus had become the leading cause of medically attended acute gastroenteritis in children during the post-rotavirus vaccine era.

### Indirect Attribution from Regression Modeling

Population-based databases that use International Classification of Disease (ICD) coding are often used to estimate trends of specific syndromes or pathogens. Those databases that are national or nationally representative can be particularly helpful in overcoming the generalizability limitations of studies performed in specific populations that may not be broadly representative. However, ICD coding for specific pathogens is typically used only when there is laboratory confirmation (*18*). Given the limited availability of direct testing for norovirus among sporadic acute gastroenteritis cases, norovirus-specific coding in these databases is insensitive and unreliable. For example, Payne et al. retrospectively retrieved ICD–9-CM discharge diagnosis codes for 278 medically attended laboratory-confirmed norovirus cases identified by active surveillance and found that none had been assigned the norovirus ICD-9-CM code (008.63) (*8*).

To overcome this issue and still use these robust sources of data, we conducted a series of modeling studies to indirectly estimate the proportion of cause-unspecified acute gastroenteritis (which represents most acute gastroenteritis–coded events) likely caused by norovirus. In brief, time-series regression models used monthly counts of acute gastroenteritis attributed to specified causes other than norovirus to estimate the number of cause-unspecified acute gastroenteritis cases likely attributable to those causes. Events attributed to these other causes and to background nonseasonal causes were subtracted from the total cause–unspecified acute gastroenteritis, and the remaining unattributed events (i.e., model residuals) were then analyzed to generate norovirus estimates. Models were developed for specific age-groups to the extent this was possible for each specific outcome.

Applying this method to national mortality data from National Vital Statistics System, Hall et al. estimated that norovirus is associated with an average of 797 deaths/year (*9*). Most (90%) of these norovirus-associated deaths and the highest mortality rate (0.20 deaths/10,000 population) occurred among persons ≥65 years of age (Figure 1, panel A). Using Healthcare Cost and Utilization Project Nationwide Inpatient Sample data, Lopman et al. estimated an average of 71,000 norovirus-associated hospitalizations each year, resulting in $493 million in health care charges (*10*). Norovirus-associated hospitalization rates exhibit a U-shaped curve (Figure 1, panel B); the highest rates occur among persons <5 years of age (9.4 hospitalizations/10,000 population) and ≥65 years of age (8.1 hospitalizations/10,000 population). To estimate rates of norovirus-associated ambulatory visits, Gastañaduy et al. applied this same method to MarketScan insurance claims databases and reported norovirus associated with 13.5 ED visits and 57.2 outpatient visits/10,000 population across all age groups (*11*). In contrast to rates of norovirus-associated mortality, rates of ambulatory visits associated with norovirus are highest in children <5 years of age (Figure 1, panels C, D). When Gastañaduy et al. extrapolated these rates to the US population, they estimated a national incidence of 399,000 ED visits and 1.7 million outpatient visits/year, corresponding to $284 million in health care charges.

**Figure 2:**
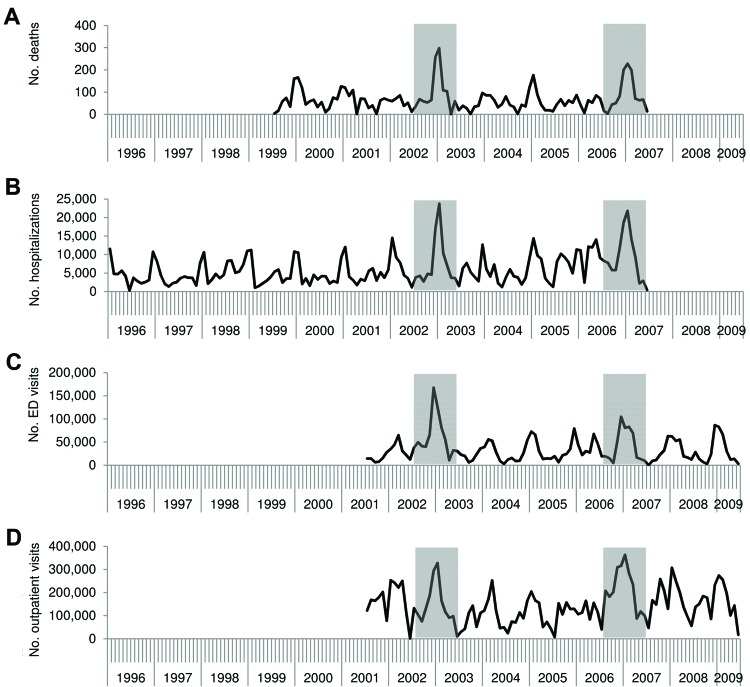
Number of A) norovirus-associated deaths, B) hospitalizations, C) emergency department (ED) visits, and D) outpatient visits across all age groups, by month and year, United States. Data were derived from studies using indirect attribution from regression modeling (*9**–**11*). Shaded areas indicate years of pandemic strain emergence (2002–2003 and 2006–2007).

Although this indirect modeling method has the potential for biases that might overestimate (e.g., assuming all residual seasonality in acute gastroenteritis is caused by norovirus) and underestimate (e.g., assuming none of the background nonseasonal incidence is associated with norovirus) norovirus incidence, it yielded temporal trends highly consistent with what is known about norovirus that help to ensure the validity of this method. These trends included a pronounced winter peak, with 63%–73% of all norovirus-associated events occurring during October–March and increases by <50% during years pandemic strains of norovirus emerged (i.e., 2002–2003 and 2006–2007) (Figure 2). These patterns have been well described through US surveillance of norovirus outbreaks (*19**–**21*) but had not been previously described among cases of sporadic norovirus illness. In addition, estimated rates of norovirus-associated outcomes from these models were generally consistent with those generated from population-based testing and attributable proportion extrapolation. For example, the all-ages outpatient rate modeled by Gastañaduy et al. (*11*) (57 outpatient visits/10,000 population) was within the uncertainty bounds of the estimate of Hall et al. (*7*) (90% credible interval 36–120 outpatient visits/10,000 population) from direct testing in the Georgia HMO population (Table 2). Likewise, the modeled hospitalization rate in children <5 years of age reported by Lopman et al. (*10*) (9.4 hospitalizations/10,000 population) was between the estimates obtained through direct testing by Payne et al. (*8*) (7.2 hospitalizations/10,000 population) and attributable proportion extrapolation by Patel et al. (*2*) (16 hospitalizations/10,000 population) (Figure 1, panel B).

## Discussion and Conclusions

**Figure 3:**
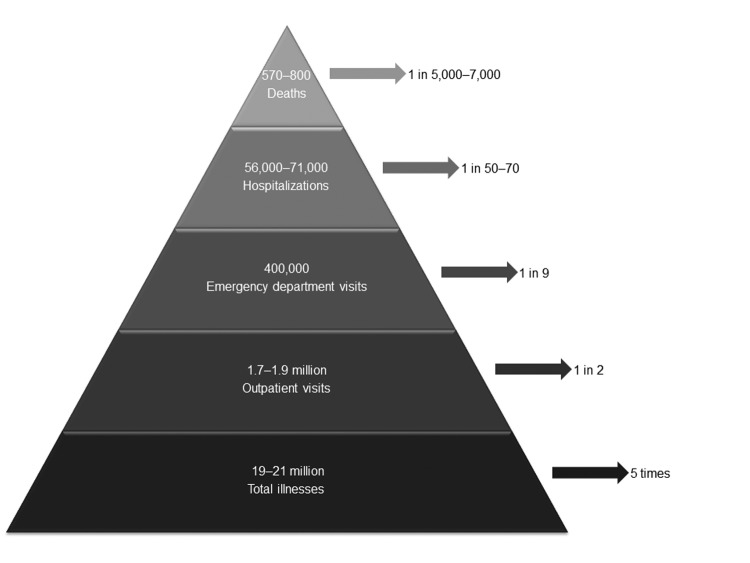
Estimates of annual burden (annual number of illnesses and associated outcomes) and individual lifetime risks for norovirus disease across all age groups, United States. Data were derived from estimates of deaths (*6**,**9*), hospitalizations (*6**,**10*), emergency department visits (*13*), outpatient visits (*7**,**11*), and illnesses (*6**,**7*). Ranges represent point estimates from different studies, not uncertainty bounds.

Over the past 5 years, substantial improvements have been made in our understanding of the burden of norovirus disease in the United States, which now represents the leading contributor to acute gastroenteritis across all age groups. By summarizing findings from studies using different methods and published over the past 5 years, we conclude that norovirus causes on average 570–800 deaths, 56,000–71,000 hospitalizations, 400,000 ED visits, 1.7–1.9 million outpatient visits, and 19–21 million total illnesses each year in the United States (Figure 3). On the basis of these rates of disease and a life expectancy of 79 years, a US resident would experience 5 episodes of norovirus gastroenteritis in his or her lifetime and an average lifetime risk for norovirus-associated outpatient visit, ED visit, hospitalization, and death of 1 in 2, 1 in 9, 1 in 50–70, and 1 in 5,000–7,000, respectively. Through age-group specific analyses, we identified that older Americans >65 years of age have the greatest risk for norovirus-associated deaths, and children <5 years of age have the highest rates of norovirus-associated medical care visits. In addition, we consistently observed across the reviewed studies increases in norovirus disease during the winter months and during years in which pandemic strains emerged.

Although the estimates summarized herein were developed by using distinct methods, each with their own strengths and limitations, the broad agreement among them is reassuring and provides a clearer picture of the norovirus disease burden in the United States. Population-based surveillance for laboratory-confirmed norovirus disease provides the most direct assessment of disease incidence, but depending on the study population, might have limited generalizability. Indirect attribution from regression modeling makes use of the most nationally representative data available but relies on temporality of acute gastroenteritis to ascribe etiology, as opposed to diagnostic testing. Attributable proportion extrapolation is somewhat of a hybrid between these 2 methods, being limited primarily by the comparability of the 2 populations involved in the extrapolation. Aside from differences in methods, the variation between estimates from the different studies might be partly caused by different time periods from which they were derived, given major year-to-year fluctuations in norovirus disease driven by the emergence of new strains.

Comparison of US norovirus incidence estimates with the few similar such estimates available from other industrialized countries showed general consistency in magnitude, especially when one considers that the uncertainty surrounding these estimates often exceeds 50% (Table 2). For example, a recent study in the Netherlands (*13*) reported a slightly higher norovirus-associated mortality rate (0.40 deaths/10,000 population) than the 2 recent US estimates (0.027 and 0.019 deaths/10,000 population) but a lower hospitalization rate (1.2 vs. 2.4 and 1.9 hospitalizations/10,000 population, respectively) (*6**,**9**,**10*). Rates of outpatient norovirus incidence from 2 studies in the United Kingdom (21 and 54 outpatient visits/10,000 population) (*14**,**15*) and 1 study in Germany (63 outpatient visits/10,000 population) (*16*) were consistent with 2 recent US estimates of 57 and 64 outpatient visits/10,000 population (*7**,**11*). Estimates of community norovirus incidence determined on the basis of 2 large-scale prospective cohort studies in the United Kingdom (470 and 450 illnesses/10,000 population) (*14**,**15*) and 1 study in the Netherlands (380 illnesses/10,000 population) (*13*) were all lower than the 2 recent US estimates (650 and 700 illnesses/10,000 population) (*6**,**7*). In contrast, a recent estimate in Canada (*17*) (1,040 illnesses/10,000 population) (*17*) was higher than estimates in the United States. However, the uncertainty bounds for the US estimates overlaps with those surrounding estimates for the United Kingdom, the Netherlands, and Canada (Table 2). Although differences in health care delivery systems and payment structures confound direct comparisons of health care visits and associated costs between countries, the substantial burden of norovirus disease is clearly not unique to the United States.

Great strides have been made in characterizing the incidence of norovirus disease in the United States; however, additional work is needed to fill some key gaps. Age-specific rates of norovirus disease, ideally from direct laboratory testing among population-based community cohorts, would help identify groups most often infected and thus those likely serving as primary human reservoirs for transmission. The causal role of norovirus and common concurrent conditions in norovirus-associated deaths also requires further clarification to help protect the most vulnerable populations. In addition, stable surveillance platforms that enable systematic and ongoing assessment of endemic norovirus disease are needed to characterize long-term trends, annual fluctuations, and effects of emergent norovirus strains.

As progress continues in the arena of norovirus vaccine development (*5*), such endemic norovirus disease data will be critical to guide formulation and quantify potential effects of vaccine. The burden of norovirus disease in the United States justifies continued efforts toward developing potential norovirus vaccines and identification of specific groups for such interventions. Our review suggests that for a vaccine to have maximal impact, it would need to demonstrate safety and effectiveness in young children and the elderly, groups at the highest risk for severe norovirus disease. Other groups at risk for epidemic disease might also include health care workers, travelers, and military personnel. Data from our review can inform cost-effectiveness and modeling studies to define an investment case and public health strategy for controlling norovirus disease in anticipation of completion of vaccine development and licensure.
